# Multi-omics analyses of glucose metabolic reprogramming in colorectal cancer

**DOI:** 10.3389/fimmu.2023.1179699

**Published:** 2023-07-05

**Authors:** Maosen Huang, Yancen Wu, Linyao Cheng, Lihua Fu, Haochao Yan, Haiming Ru, Xianwei Mo, Linhai Yan, Zijie Su

**Affiliations:** ^1^ Guangxi Clinical Research Center for Colorectal Cancer, Guangxi Medical University Cancer Hospital, Nanning, Guangxi Zhuang Autonomous Region, China; ^2^ Department of Gastrointestinal Surgery, Guangxi Medical University Cancer Hospital, Nanning, Guangxi Zhuang Autonomous Region, China; ^3^ Department of Research, Guangxi Medical University Cancer Hospital, Nanning, Guangxi Zhuang Autonomous Region, China

**Keywords:** multi-omics, glucose metabolic reprogramming, colorectal cancer, immunology, metastasis

## Abstract

**Background:**

Glucose metabolic reprogramming (GMR) is a cardinal feature of carcinogenesis and metastasis. However, the underlying mechanisms have not been fully elucidated. The aim of this study was to profile the metabolic signature of primary tumor and circulating tumor cells from metastatic colorectal cancer (mCRC) patients using integrated omics analysis.

**Methods:**

PET-CT imaging, serum metabolomics, genomics and proteomics data of 325 high 18F-fluorinated deoxyglucose (FDGhigh) mCRC patients were analyzed. The para-tumor, primary tumor and liver metastatic tissues of mCRC patients were used for proteomics analysis.

**Results:**

The glucose uptake in tumor tissues as per the PET/CT images was correlated to serum levels of glutamic-pyruvic transaminase (ALT), total bilirubin (TBIL), creatinine (CRE). Proteomics analysis indicated that several differentially expressed proteins were enriched in both GMR and epithelial-mesenchymal transition (EMT)-related pathways. Using a tissue-optimized proteomic workflow, we identified novel proteomic markers (e.g. CCND1, EPCAM, RPS6), a novel PCK1-CDK6-INSR protein axis, and a potential role for FOLR (FR) in GMR/EMT of CRC cells. Finally, CEA/blood glucose (CSR) was defined as a new index, which can be used to jointly diagnose liver metastasis of colorectal cancer.

**Conclusions:**

GMR in CRC cells is closely associated with the EMT pathway, and this network is a promising source of potential therapeutic targets.

## Introduction

Glucose metabolic reprogramming (GMR) of cancer cells, also known as the Warburg effect, is defined as the increased rate of glycolysis and glucose consumption even in the presence of oxygen, which fulfils the high energy and biosynthetic demands of the rapidly proliferating tumor cell (1). Studies are increasingly recognizing Warburg effect as an oncogenic feature associated directly with dysfunctional mitochondrial respiration. Positive-emission tomography (PET) has demonstrated 20~30 higher glycolytic ability of several tumor cells compared to normal cells, which correlates with faster growth and greater invasiveness of the tumor (2).

Another hallmark of cancer progression and metastasis is the epithelial-mesenchymal transition (EMT), wherein the tumor cells lose their epithelial characteristics, and attain the mesenchymal phenotype with greater migratory and invasive capacities. It is the key process driving metastasis and malignant progression of tumors. Recent studies have linked EMT with the metabolic reprogramming of tumors, indicating that it likely regulates metabolism of cancer cells (3, 4). Little is known regarding the metabolic alterations in CRC cells, especially the glucose-related pathways, during EMT. The aim of this study was to characterize the metabolic reprogramming in CRC cells undergoing EMT in order to identify the metabolic pathways involved in cancer progression. To this end, we analyzed the 2-deoxy-2-[18F] fluoro-glucose (18F-FDG) PET-CT images and serum metabolites in 325 metastatic CRC (mCRC) patients, and explored between the epithelial-mesenchymal transition and glucose metabolic reprogramming (EMT-GMR) network by evaluating the genomics and proteomics data of paired primary tumor, para-tumor and liver metastatic tissues. Finally, we propose CEA/blood glucose ratio(CSR) as a new indicator, which can be used to jointly diagnose liver metastasis of colorectal cancer.

## Materials and methods

### Samples collection

We retrospectively collected the electronic medical record data of colorectal cancer patients with and without liver metastasis from January 2015 to December 2020. The patient was diagnosed with colorectal cancer through the initial surgical resection or pathological examination. Data were preoperative and without any treatment or surgery. The clinical data of participants were all from the Guangxi Medical University Cancer Hospital, including demographic data, serological test data and PECT imaging data. Patients with colorectal cancer were split into discovery cohort and validation cohort and tested the clinical practicability of CSR on two datasets.

### Whole-exome sequencing

Whole-exome sequencing for all patients were performed on Illumina Novaseq 6000 using 2 × 150 bp pair-end sequencing method, according to the manufacturer’s instructions. Tumor sample was obtained *via* core biopsy during initial diagnostic procedure. Genomic DNA was extracted from paraffin embedded slides by using an E.Z.N.A tissue DNA Kit (Omega Biotek, Doraville, USA) according to the manufacturer’s protocol.Cell-free DNA (cfDNA) was extracted as described. In brief, 10 ml of whole blood was collected and cfDNA was extracted from plasma samples using the QIAamp Circulating Nucleic Acid kit (Qiagen) according to the manufacturer’s instructions.Library preparation was performed as described. Fragments of size 200–400bp were selected and followed by hybridization with capture probes baits, hybrid selection with magnetic beads and PCR amplification. Indexed samples were sequenced on Hiseq Xten VS NovaSeq 6000 (Illumina, Inc., USA) with 2 × 150 bp pair-end reads. The sequencing data in the FASTQ format were mapped to the human genome (hg19) using BWA aligner 0.7.10. Local alignment optimization, variant calling and annotation were performed using GATK 3.2, MuTect 2, and VarScan, respectively.

### Protein chromatography and mass spectrometry (NanoLC-ESI-MS/MS analysis: DIA+PRM)

After protein extraction, the tissue was subjected to SDS-PAGE electrophoresis and Coomassie Brilliant Blue staining for 1h, and decolorized with decolorizing solution. The protein band should be clear. Compared with the control group, the treatment could see the difference band. Subsequently, the protein is subjected to reduction, alkylation, acetone precipitation, cleaning, and enzymatic digestion for proteolysis. Take out the enzymatically hydrolyzed sample, select a suitable desalination column according to the experimental requirements for desalination and perform peptide quantification. Finally, by establishing a spectrogram database and collecting and analyzing data. Subsequently, protein annotation was performed, including GO and KEGG annotation. All analyses were performed by a Triple TOF 5600 mass spectrometer (SCIEX, USA) equipped with a Nanospray III source (SCIEX, USA). The MS/MS data were analyzed for protein identification and quantification using ProteinPilot software (v.5.0) (Online **Supplementary Methods**). In addition, Ca represents colorectal cancer tissue, P represents adjacent tissue, and L represents liver metastasis tissue of colorectal cancer. Among them, Ca: P represents the comparison between cancer tissue and adjacent tissue, Ca: L represents the comparison between cancer tissue and metastatic tissue, and P: L represents the comparison between adjacent tissue and metastatic tissue.

### Statistical analysis of data

The data were analyzed by SPSS software (SPSS 17.0, Chicago, Illinois, USA) and Graphpad Prism Version 7.0 software (LA Jolla Ca 92037, USA). Nonparametric test was used to test the difference between the two groups, P < 0.05 was considered statistically significant. Two groups of data are continuous numerical variable data, using the mean ± standard error of mean (SEM) for the statistical description. Receiver operating characteristic (ROC) curve was utilized to explore the diagnostic ability of related indicators in colorectal cancer patients with liver metastasis and without liver metastasis. Its diagnostic ability was determined by area under the curve (AUC). The value of the cutoff is calculated according to the Youden index (sensitivity + specificity - 100%). Therefore, the sensitivity and specificity under the considerable cutoff value can be known and can be used for clinical reference.

## Results

### CRC has a distinct metabolomics profile

**Figure 1:**
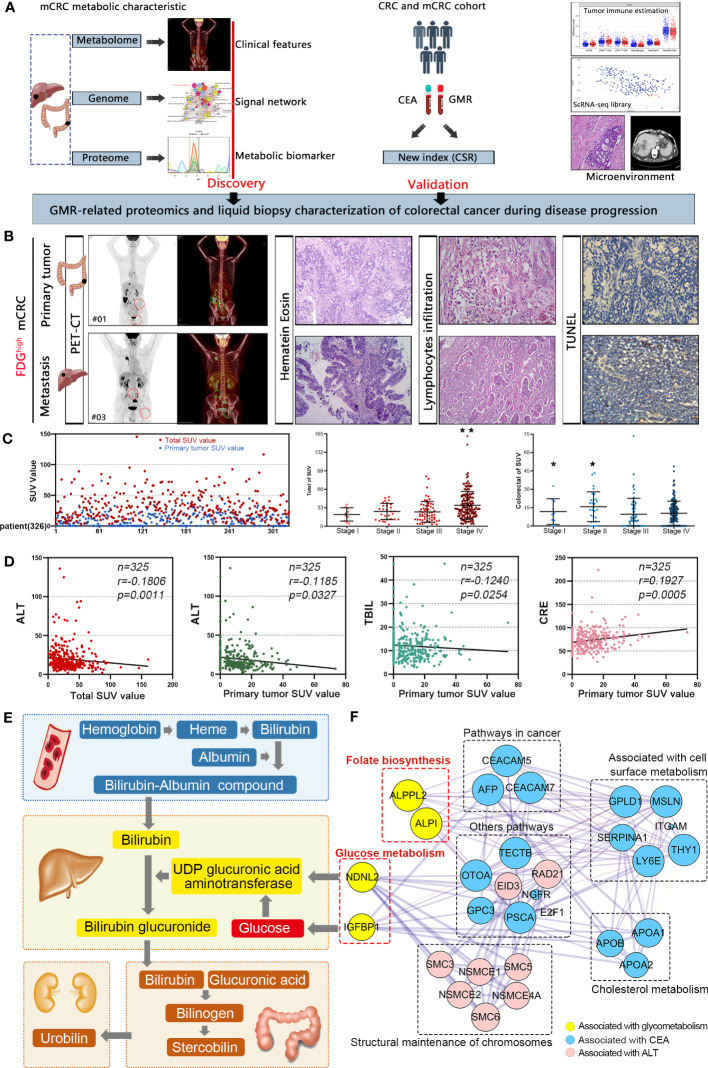
Colorectal cancer is characterized by distinctive clinical metabolomics composition. **(A)** A scheme showing experiments and integrated analyses which were performed. **(B)** The PET-CT, HE, LI, and TUNEL of typical samples. **(C)** 18F-FDG standard uptake value in mCRC patients. *p<0.05, **p≤0.01. **(D)** SUV was positively correlated with CEA, ALT, total bilirubin and creatinine. **(E)** Interaction network of glucose metabolism in liver, kidney, colorectal and other parts. **(F)** Molecular interaction networks of glucose metabolism in colorectal cancer.

Explore the interactions between glucose metabolism networks and colorectal cancer (**Figure 1A**). We have obtained PET/CT information from 325 CRC patients, which can to some extent reflect the overall glucose metabolism of colorectal cancer patients. At the same time, we also collected and analyzed pathological section information of these patients’ tissues, including HE staining, lymphocyte infiltration (LNI), and tumor apoptosis index (TUNEL). We found that an 18F-FDG standard uptake value (SUV more than 8.0) of 18F-FDG was 100% suitable for the diagnosis of primary and metastatic tumors. However, glucose metabolism in tumor tissue did not directly reflect lymphatic infiltration around the tumor and the degree of apoptosis in the tumor tissue (**Figure 1B**). We also explored the relationship between known carcinogenesis proteins and glucose metabolism. These markers were not directly associated with the glucose metabolism of tumors. The regulation network of glucose metabolism in tumors is more complex than our understanding of it (**Supplementary Table 1**, **Supplementary Figures 1A, B**). Furthermore, we conducted a SUV analysis and found that the SUV after the occurrence of distant metastasis (Stage VI) was significantly higher than that at the other three stages, and was mainly distributed in the 29.42-34.38 interval, but did not increase indefinitely. Moreover, we observed that patients at stage I-II had a higher SUV in the colon than stage III-IV patients, indicating that glucose metabolism was higher during the early stage of tumor growth (**Figure 1C**). To understand the relationship between glucose metabolism and other serum metabolites, we analyzed serological data on the patients. SUV was positively correlated with levels of CEA, ALT, total bilirubin and creatinine (P < 0.05), reflecting interactions in the glucose metabolism in the liver, kidney, colon and other organs (**Figure 1D**, **Supplementary Figure 1C**, **Supplementary Table 2**). Based on these results, we found that glucose participates in the biosynthesis of glutamic transaminase through the glucuronate pathway. In our study, CEA and ALT levels were strongly associated with SUV. However, previous studies have found that IGFBP1 can promote glucose synthesis. In addition, ALPPL2 and ALP1 are involved in folate biosynthesis. Previous studies have shown that folate metabolism can also indirectly affect the glucose metabolism pathway (**Figures 1E, F**). Conclusively, these results indicated that changes in metabolism are associated with colorectal cancer, especially for glucose metabolism, which is regulated at different levels and by various organs.

### Genetic networks of GMR in CRC

**Figure 2:**
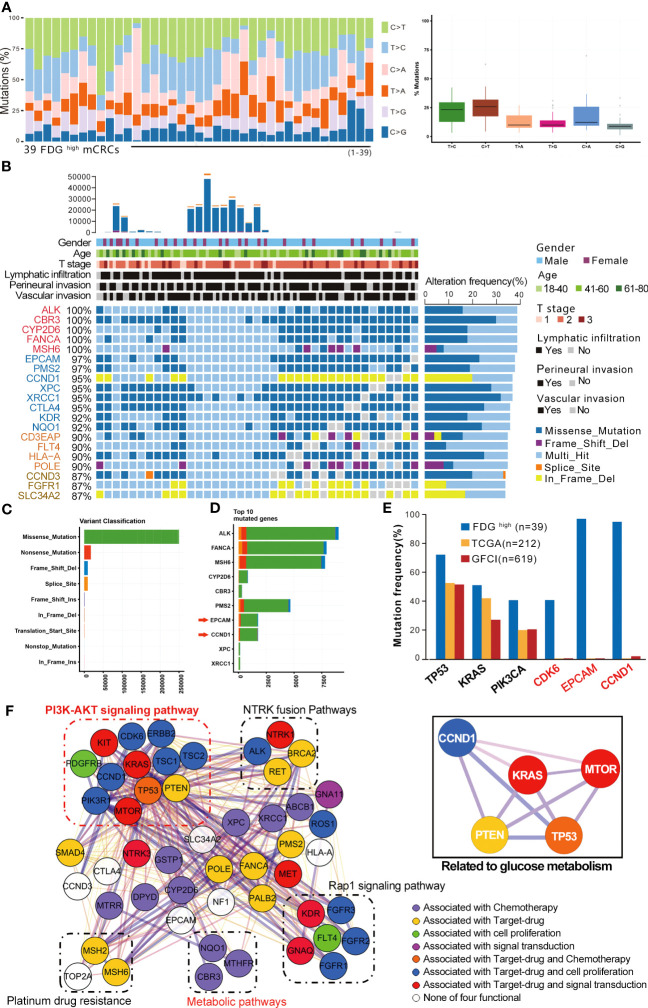
Glucose metabolic reprogramming genetic networks of CRC patients. **(A)** FDG ^high^ mCRC patients were selected for mutant gene characteristics analysis. **(B)** Comparison of all frequently mutated genes. **(C)** Variant classification of mutated genes in primary tissues of CRC. **(D)** Comparison of the top 10 frequent SCNAs in primary tissues of CRC. **(E)** Comparison of frequently mutated genes in FDG ^high^, TCGA and GFCI cohorts. **(F)** Interaction network of glucose metabolism in liver, kidney, colorectal and other parts.

To identify the genes involved in GMR during the malignant transformation of the enterocytes to adenocarcinoma cells, we analyzed the WES data of 39 FDG ^high^ mCRC patients and screened for the differentially expressed genes (DEGs) related to metabolic functions (**Supplementary Table 3**). As shown in **Figure 2A**, there were 344 mutated DEGs and the predominant mutations were the C > T and C > T transversions. There were no obvious differences in the mutations among the putative driver genes or with mCRC datasets from previous studies (**Supplementary Figures 2A-F**) (5, 6).The mutation rate of the top 20 genes exceeded 85% (**Figure 2B**). Furthermore, CCND1 and EPCAM were correlated to the KEGG pathways of nutrient metabolism and epithelial-mesenchymal transition (**Figures 2C, D**). The highly mutated genes were further analyzed in the cBioPortal for Cancer Genomics, GFCI (Cell Reports 2016, n=619) and TCGA (Nature 2012, n=212) databases. As shown in **Figure 2E**, CDK6, EPCAM and CCND1 are frequently mutated in CRC, and mutations rates in TP53, KRAS and PIK3CA are also high in the FDG ^high^ mCRC patients. Mutations in KRAS, TP53, PTEN and MTOR promote tumor development by inducing multiple metabolic pathways (7, 8). Therefore, we hypothesized that mutated CCND1 also affects glucose metabolism in CRC similar to KRAS mutations (**Supplementary Figure 2G**). To support this hypothesis, we constructed a protein-protein interaction (PPI) network of 50 highly mutated genes (**Figure 2F**). CCND1, KRAS, TP53, MTOR and PTEN showed high interaction scores (0.809-0.997, **Supplementary Table 4**), and were all enriched in the PI3K-AKT signaling pathway. We next predicted the relationship between CCND1 DNA copy number changes and infiltration of immune cells. As shown in **Figure S2H**, arm-level gain significantly increased CD8+T cell and Neutrophil. The Pfam database further revealed 15 mutations in the CCDN1 protein sequence, most arising from missense mutations. The T286I mutations was in particular an oncogenic hotspot (**Supplementary Figure 2I**). In addition, high copy numbers and overexpression of CDK6, EPCAM and CCND1 correlated with poor disease free survival (DFS) and overall survival (OS) (**Supplementary Figure 9**), which was consistent with previous studies (GSE12945, 17536, 17537). Taken together, these findings indicated that CCND1 is frequently mutated in mCRC and possibly involved in GMR.

### Protein networks of GMR

**Figure 3:**
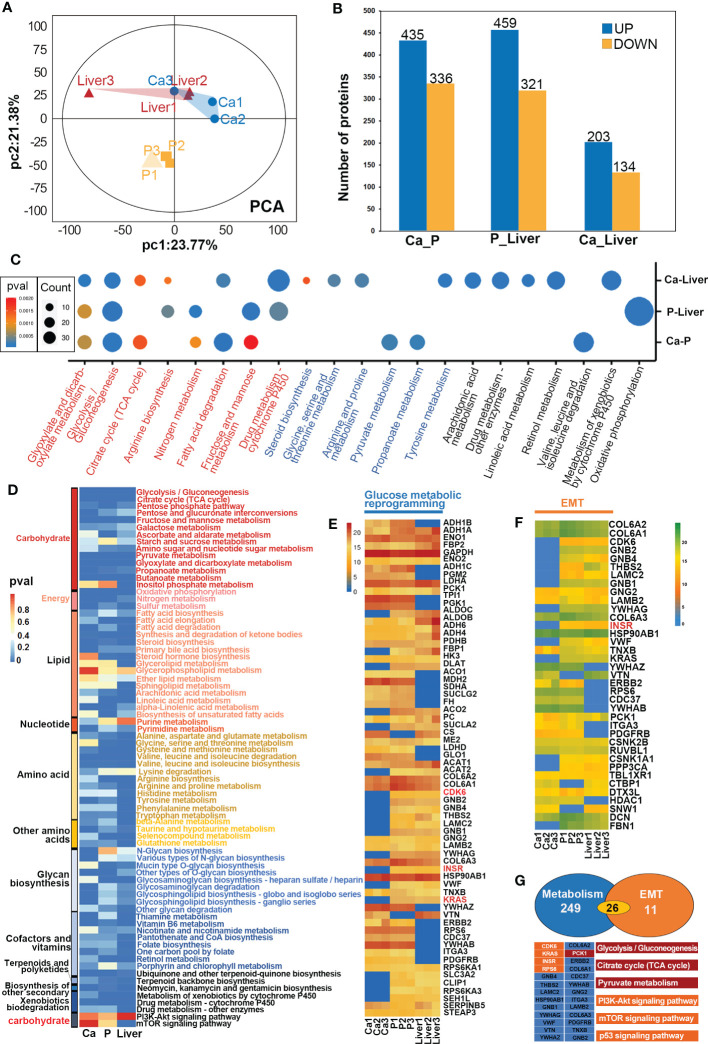
GMR based on proteomic data. **(A)** Principal-component analyses (PCA) of protein in paired Ca, P and Liver groups. **(B)** Expression levels of DEPs between different groups. **(C)** Heat map of all protein DEPs in the proteomic function analysis. **(D)** The heatmap shows the significance of cellular processes being enriched by proteins in different groups. **(E)** The heatmap shows the significance of GMR-related proteins in different groups. **(F)** The heatmap shows the significance of EMT-related proteins in different groups. **(G)** Crosstalk proteins between GMR and EMT.

**Figure 4:**
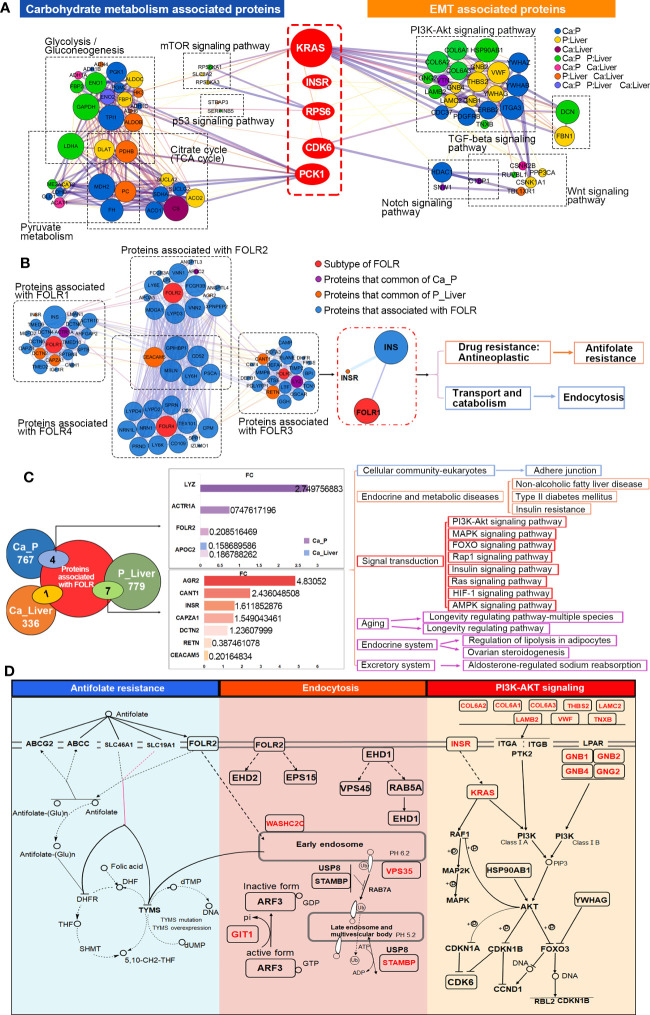
Key DEPs related to GMR and EMT co-participate. **(A)** Functional enrichment analysis to construct key DEPs interactions. **(B)** Interaction network analysis for all 4 subtypes of FOLR. **(C)** Integrated analysis of 40 EMT-related proteins. **(D)** Overview of signaling pathways based on integrated proteogenomic analysis.

The proteins associated with GMR in CRC were identified by comparing the proteomic alterations in paired Ca, P and Liver samples from 12 mCRC patients (**Supplementary Figure S8**) using data independent acquisition (DIA) analysis. The distribution of proteins identified on the basis of molecular weight and peptide number is shown in **Supplementary Figure 3B**. Principal component analyses (PCA) revealed distinct protein expression patterns of the different tissues (**Figures 3A**, **4A**), and hierarchical clustering analyses also revealed significant differences in protein expression levels among the different groups (**Supplementary Figures 3C, D**, **4B**). We identified 771 differentially expressed proteins (DEPs) in the Ca samples relative to P, of which 435 were upregulated and 336 were downregulated. In addition, 780 DEPs were detected in P tissues relative to the Liver, of which 459 were upregulated and 321 were downregulated. Finally, there were 337 DEPs in Ca versus Liver tissues, including 203 and 134 up and downregulated proteins respectively (**Figure 3B**). The mismatch repair-related protein RFC5 was up-regulated in Ca vs P and the pancreatic secretion-related protein CLCA1 was up-regulated in P vs Liver group. GO enrichment analysis of the DEPs in Ca_P and Ca_Liver showed that poly(A) RNA binding was common to both groups. Furthermore, KEGG enrichment analysis showed that 43% of the top 20 pathways in the three comparison pairs, and 70% in the Ca_Liver comparison, were related to GMR (**Supplementary Figures 4C, D**). The glyoxylate and dicarboxylate metabolism and glycolysis/gluconeogenesis pathways were enriched in all pairs (**Figure 3C**), and the common DEPs were significantly enriched in glycolysis/gluconeogenesis, citrate cycle (TCA cycle) and pyruvate pathways that are associated with glucose metabolism (**Figure 3D**). These results strongly indicate that GMR is initiated in the early stages of CRC and persists during tumor progression and metastasis. A total of 66 DEPs across all comparison pairs were enriched in the three glucose metabolism pathways (**Figure 3E**). Moreover, PCK1, KRAS, INSR, CDK6, and RPS6 were also involved in PI3K-AKT signaling pathway. Furthermore, we integrated proteomics data of EMT-related proteins from 4 major signaling pathways (**Figures 3F, G**), and found these proteins exert effects on EMT by regulating DNA repair, angiogenesis, cell proliferation, apoptosis, glycolysis/gluconeogenesis, protein synthesis, cell cycle and proteolysis. The Notch signaling pathway related-proteins were expressed higher in Ca_P than in Ca_Liver, however, the TGF-beta signaling pathway related-proteins were expressed low in Ca_P than in Ca_Liver. For most proteins were enriched in PI3K-Akt pathway, it is the most complex to regulate, and proteins changes is inconsistent (**Supplementary Figure 3A**).

### Key DEPs related to GMR and EMT co-participate

To further elucidate the mechanisms underlying the interactions between GMR and EMT, we screened for DEPs common to the pathways enriched in both processes. In the MCODE complex/subnetwork, the PI3K-Akt signaling pathway overlapped with glycolysis/gluconeogenesis, citrate cycle (TCA cycle) and pyruvate metabolism *via* PCK1, as did the mTOR and p53 signaling pathways *via* KRAS/INSR/RPS6/CDK6 axis (**Figure 4A**). Both the occurrence and metastasis of CRC are highly associated with GMR and EMT, which suggests GMR/EMT-related proteins have the potential as therapeutic targets. Interestingly, we have noted the expression of FOLR is not influenced by EMT transformation, and found that FOLR1 and FOLR2 were enriched in the same pathway associated with the antifolate resistance and endocytosis using interaction network analysis between 4 subtypes of FOLR, moreover, FOLR1 and FOLR2 were found expressed in the same location (**Figure S5**). Otherwise, we found FOLR1 was interacted with INSR through INS (**Figure 4B**). Among the DEPs between Ca_P and normal sample, four proteins have interaction with FOLR, including LYZ, ACTR1A, FOLR2 and APOC2. Among the DEPs between Ca_P and Ca_Liver, seven proteins have interaction with FOLR, including AGR2, CANT1, INSR, CAPZA1, DCTN2, RETN and CEACAM5. Among the DEPs between Ca_Liver and normal sample, only APOC2 was found interacted with FOLR. FOLR2 was including PI3K-Akt signaling pathway, *via* regulating its downstream gene INSR (**Figure 4C**). In conclusion, FOLR2 contributes to the occurrence and development of mCRC directly or indirectly, and the main enriched pathways analysis of FOLR2 and CRC was plotted in **Figure 4D**. In brief, FOLR regulated GMR/EMT *via* RI3K-AKT pathway in the whole process of CRC.

### PCK1-CDK6-INSR axis correlated with FOLR-related GMR and EMT

**Figure 5:**
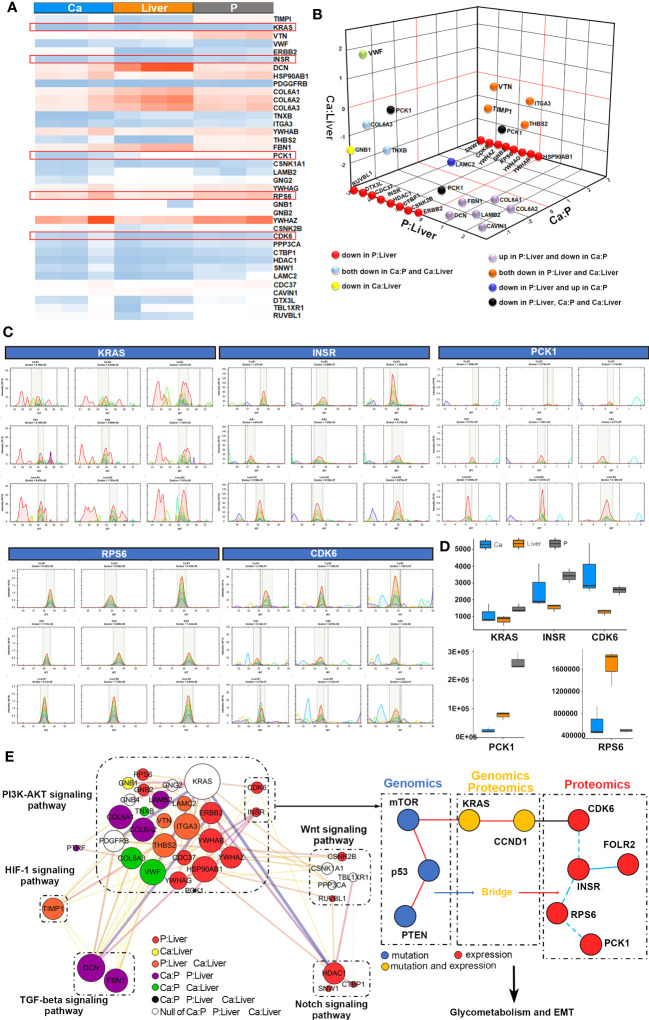
PCK1-CDK6-INSR axis correlated with protein marker of FOLR-related GMR and EMT. **(A)** The heatmap shows the significance of EMT/GMR-related proteins in different groups by PRM. **(B)** Relationship of differentially expressed proteins among Ca-Liver, Ca-P and P-Liver by PRM. **(C, D)** Peak Diagram of the target peptide, including KRAS, INSR, PCK1, RPS6 and CDK6. **(E)** Overview of PCK1-CDK6-INSR axis based on PRM analysis.

The FOLR2-CDK6-INSR axis was further analyzed by LC-MS/MS in the PRM mode (**Supplementary Figure 6A**). All the quantified peptides exhibited an excellent linear fit between the observed retention time and iRT in the library. The retention time of inner-label iRT was stable and the error was small (**Supplementary Figure 6B**), indicating reliable peptide identification. We selected 40 DEPs enriched in PI3K-Akt, Wnt, Notch and TGF-beta signaling pathways (**Figure 5A**), and found significant differences in the expression levels of targeted proteins between the different comparison pairs, especially the 5 key DEPs. As shown in **Figure 5B**, PCK1 levels were significantly different in all three groups in PRM, whereas INSR, CDK6 and RPS6 showed significant differences between P and Liver samples. The peptide peaks of INSR, CDK6 and RPS6 respectively had higher quantitative values of b and y ions in the Liver compared to P, indicating that these proteins are upregulated during metastasis. Interestingly, PCK1 showed significant differences only in the Ca_P and Ca_Liver pairs in DIA, and that for KRAS was observed in P_Liver (**Figures 5C, D**, **Supplementary Figure 6C**). Based on both DIA and PRM results, we surmise that PCK1, CDK6, KRAS, INSR and RPS6 are the key proteins synergizing EMT and GMR. Taken together, mutations in MTOR, TP53, PTEN, KRAS and CCND1 are conducive to GMR during EMT, and mutations in KRAS and CCND1 in particular upregulates the FOLR2-CDK6-INSR-RPS6-PCK1 axis (**Figure 5E**).

### Functional analysis of the FOLR-related proteins

**Figure 6:**
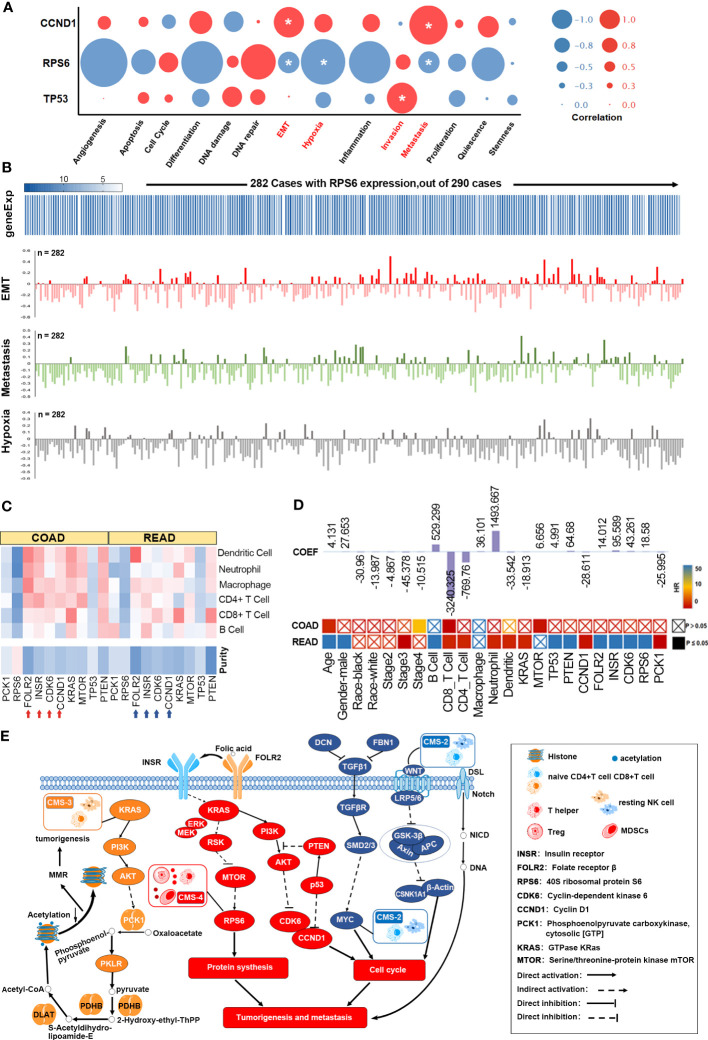
Functional analysis of the FOLR-related proteins. **(A)** GMR/EMT-related key DEPs of functional states in TIMER database. **(B)** RPS6 is directly related to the function of GMR, EMT, and metastasis. **(C)** The correlation of 6 DEPs expression and 5 key mutant gene with immune infiltration level. **(D)** A multivariable cox proportional hazard model including clinical factors (age, gender, ethnicity, tumor stages), gene and protein expression (FOLR2, INSR, CDK6, RPS6, PCK1, KRAS, MTOR and PTEN and TP53). **(E)** The FOLR-related proteins are correlated with microenvironmental characteristics.

To further elucidate the potential role of synergistic GMR/EMT in tumor heterogeneity, we analyzed the-omics data at the single cell level using the GSE81861 dataset from CancerSEA, which includes the data of 290 single cells (endothelial, epithelial, fibroblast) from 11 CRC patients. The correlation between 5 key DEPs (KRAS, INSR, CDK6, RPS6 and PCK1), 5 key mutant gene (KRAS, CCND1, PTEN, MTOR and TP53) and 14 functional states (occurrence, metastasis, treatment etc.) of CRC (**Supplementary Figure 7A**) were analyzed. CCND1 was significantly correlated to EMT (Cor=0.208, p=0.032) and metastasis (Cor=0.265, p=0.006), TP53 to invasion (Cor=0.207, p=0.02), and RPS6 to EMT (Cor=-0.147, p=0.013), hypoxia (Cor=-0.307, p=0.0001) and metastasis (Cor=-0.148, p=0.013) (**Figure 6A**). RPS6 expression was detected in 282 cells and correlated to the aforementioned functions (**Figure 6B**). Likewise, functional correlation of TP53 and CCND1 was established in 290 cells (**Supplementary Figure 7B**). T-SNE analysis further revealed uniform distribution of CCND1, PTEN, MTOR, TP53, INSR, CDK6, RPS6 and PCK1 (**Supplementary Figure 7C**).

TIMER database was then used to analyze the correlation of 6 DEPs and 5 key mutant gene with the extent of immune infiltration, the results showed that FOLR2, INSR, CCND1, KRAS and PTEN, highly expressed in the microenvironment in colon adenocarcinoma (COAD) and rectum adenocarcinoma (READ), were negatively correlated with tumor purity, and was positively correlated with immune infiltration (**Figure 6C**). Mutations in KRAS and TP53 reduced the infiltration of macrophages and neutrophils, while mutated PTEN was associated with increased infiltration of B cells, CD8+ T cells and macrophages (**Supplementary Figure 7D**). Multivariate Cox regression analysis was performed with age, gender, race, stage, B cells, CD8+ T cells, CD4+ T cells, macrophages, neutrophils, dendritic cells, KRAS, MTOR, TP53, PTEN, CCND1, FOLR2, INSR, CDK6, RPS6 and PCK1 as the covariates. Age (HR=1.044, p=0.001), stage 4 (HR=6.413, p=0.003), CD8+ T cells (HR=0.002, p=0.032) and MTOR (HR=0.509, p=0.029) were the independent prognostic factors of COAD (263 patients with 63 deceased). For READ (82 patients with 14 deceased), all except Caucasian race, stage 2 and 4, macrophage infiltration and MTOR were significantly associated with survival (**Figure 6D**). In other words, tumor immune cells expressing GMR/EMT-related factors are prognostically relevant in CRC (**Supplementary Figure 7E**). According to our prognostic model (**Figure 6E**), the molecular subtypes of CRC (CMS 1, 2, 3 and 4) were also highly correlated with the immune microenvironment. The CMS2 tumors displayed strong upregulation of WNT and MYC that are implicated in CRC genesis. CMS3 tumors had frequent KRAS mutations and disrupted metabolic pathways. CMS2 and CMS3 tumors with intermediate prognosis exhibited low immune and inflammatory signatures. In contrast, CMS4 tumors showed clear upregulation of genes associated with EMT, angiogenesis, matrix remodeling and complement inflammatory system. Finally, CMS4 tumors also showed increased infiltration of lymphoid and myeloid cells and exhibited poor prognosis.

### Validation of GMR/EMT-related biomarkers in CRC

**Figure 7:**
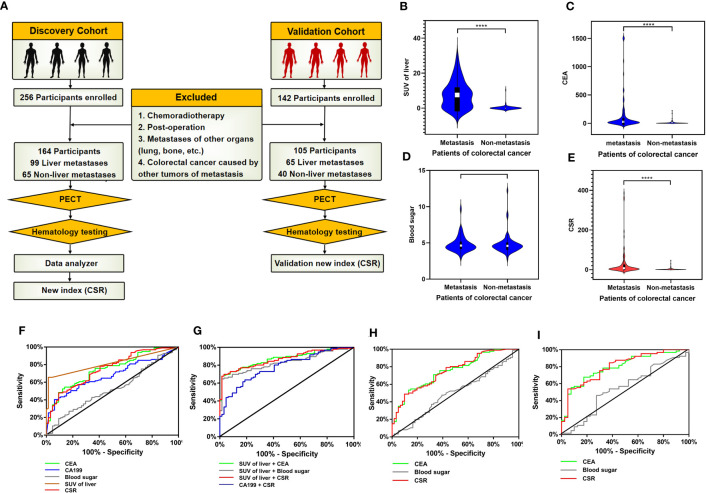
Validation of GMR/EMT-related biomarkers in CRC. **(A)** Flow chart designed of the study: the cohort in this study was consistsed of discovery cohort and validation cohort (including colorectal cancer patients with liver metastasis and without liver metastasis). In the discovery cohort, the data of SPECT and serology were collected, and the combined index of CSR (CEA to blood sugar ratio) was statistically analyzed. **(B–E)** Analysis of clinical data of discovery cohort: Comparison SUV of liver in SPECT, CEA, blood glucose and CSR levels of serology between colorectal cancer patients with liver metastasis and without liver metastasis. The data were presented as means ± SEM, **** *p* < 0.0001. **(F)** The diagnostic ability of single CEA, CA199, blood sugar, liver SUV and CSR in differentiating liver metastatic colorectal cancer. **(G)** ROC curve analysis: the diagnostic ability of combined indicators in differentiating liver metastatic colorectal cancer were SUV of liver + CEA, SUV of liver + blood sugar, SUV of liver + CSR and CA199 + CSR, respectively. **(H)** ROC curve of CEA, CSR and blood sugar in discovery cohort excluding diabetes mellitus patients. **(I)** ROC curve of CEA, CSR and blood sugar in validation cohort.

We retrospectively analyzed the clinical data of 398 patients with colorectal cancer diagnosed by PET (**Figure 7A**). Patients in the discovery cohort were divided into 99 patients with liver metastasis group and 65 patients without liver metastasis group. The demographic characteristics, CEA, fasting blood glucose and CSR levels were presented in **Supplementary Table 5**. Age and gender of these two groups were comparable. Our data showed that the SUV and CEA were significant difference between the two groups (P < 0.0001), yet there was no significant difference in blood glucose with these two groups (**Figures 7B–D**). It is worth noting that only considering the blood glucose was meaningless in the progression of metastasis. Considering jointly the significant impact of CEA produced by the primary tumor on the occurrence of colorectal cancer, our data found that CSR has significant statistically difference in patients with and without liver metastases (p<0.0001) (**Figure 7E**). More importantly, CEA was 128.9 ± 30.15 in colorectal patients with liver metastasis and 27.43 ± 6.899 in CSR patients with liver metastasis. It is forecasted that the interaction between the two factors possibly lead to tumor metastasis, which is worthy of further exploration. Based on the above exploration, we assessed the diagnostic potential of CSR as a biomarker in 164 patients with metastatic colorectal cancer from discovery cohort. The ROC curve was used to evaluate the diagnostic ability of CSR between liver metastasis and without liver metastasis. We also compared single CEA, CA199, SUV of liver and other common clinical indicators between the two groups (**Figure 7F**) with areas under the ROC curve (AUC): 0.748 (95%CI 0.673-0.822), 0.676 (95%CI 0.595-0.757) and 0.815 (95% CI 0.750-0.880), respectively (**Supplementary Table 6**). However, the cutoff of CSR was 5.025, AUC: 0.753 (95%CI 0.678-0.828), sensitivity: 47.92%, and specificity: 90.63%. Therefore, we further combined CSR with CEA, CA199 and liver SUV to joint analysis (**Figure 7G**). As shown in **Supplementary Table 6**, the AUCs of combined detection of CSR+liver SUV were 0.878 (95% CI 0.813-0.922), which was higher than that of combined detection of CEA + liver SUV (AUC = 0.871 (95% CI 0.818-0.925), and the sensitivity and specificity of CSR + liver SUV group was 67.79% and 96.88% respectively.

Based on the characteristics of high glucose metabolism in colorectal cancer patients in the discovery cohort, we further investigated whether CSR was influenced by diabetes mellitus. We excluded the influence of diabetes mellitus and 17 patients with type II diabetes were finally excluded. The remaining patients were 86 liver metastasis group and 61 non-liver metastasis group. According to ROC curve, AUCs of CSR were 0.7528, sensitivity was 48.84% and specificity was 86.89% (**Figure 7H**, **Supplementary Table 7**). Compared with cohort data without excluding diabetes mellitus patients, the results were roughly similar, and the change of CSR displayed a minor change. Therefore, it considered that CSR had certain stability.

The above discovery cohort study has explained the feasibility of CSR. To further enhanced the repeatability and clinical practicability of CSR, we collected another batch of 142 colorectal cancer patients from January 2017 to December 2022 to further validate CSR and defined these patients as validation cohort (**Figure 7A**). After screening according to the exclusion criteria, a total of 105 patients entered our validation cohort, including 65 colorectal cancer patients with liver metastasis and 40 colorectal cancer patients without metastasis. Demographic data and serological clinical indicators of these colorectal cancer patients were collected, and the age and gender of these patients were comparable (**Supplementary Table 8**). Through our data analysis, CEA and CRS still had significant statistical differences between liver metastasis group and non-liver metastasis group (P < 0.001). In addition, ROC curve analysis of 105 participants showed that AUC of CEA was 0.799 (95% CI 0.713-0.884), sensitivity was 67.69%, and specificity was 82.50%. The AUC of CSR was 0.804 (95% CI 0.719-0.889), the sensitivity was 53.85%, and the specificity was 95.00% (**Figure 7I**, **Supplementary Table 9**).

## Discussion

Tumor metabolic reprogramming manifests as a significant increase in glycolysis, glucose uptake and consumption, lipid and protein synthesis, and catabolism of amino acids such as glutamine. The acidic microenvironment resulting from excessive glycolysis is highly conducive to tumor growth, invasion and immune evasion. The aberrant tumor cell metabolism is in turn promoted by several key oncogenic signaling pathways to support their growth and survival. Furthermore, some metabolic alterations are crucial for malignant transformation.

KRAS mutations are closely associated with the Warburg effect, and promote glucose uptake by upregulating the glucose transporter 1 (GLUT1) (9). In addition, the PI3K pathway also increases glucose uptake and desensitizes cells to hyperglycemic conditions by activating GLUT1 (10). The altered glycolytic state is driven by changes in the enzymes involved in glucose metabolism contributed to CRC progression (11). The TCA enzyme pyruvate dehydrogenase B (PDH) was upregulated in the para-tumor and primary tumor tissues relative to the liver metastases led to the accumulation and subsequent extrusion of lactate into the extracellular matrix (ECM) (12). The resulting acidosis promotes degradation of ECM proteins like collagen, laminin, fibulin and proteoglycan, which promotes the invasion and metastasis of cancer cells. Low tumor pH can also render exogenous alkaline anti-cancer drugs ineffective, and promotes immune evasion of tumor cells by repressing lymphocyte activity and proliferation, which is consistent with our prediction model (13).

EMT is regulated by several development-related transcription factors. For instance, TWIST induces the expression of mesenchymal markers and inhibits the epithelial protein E-cadherin during EMT (14). A recent study showed that overexpression of TWIST increases glucose utilization and lactate production by upregulating glycolytic genes In addition (15), HIF-1α induces the expression of EMT regulators such as Snail and TWIST, and binds to the hypoxia-response element (HRE) in the TWIST proximal promoter (16). Thus, key effectors of EMT also play a role in regulating tumor metabolism. Conversely, we found that the key proteins involved in dysregulated metabolism also promote EMT. For instance, the PI3K-AKT signaling pathway concurrently mediates GMR and EMT of CRC cells by targeting common proteins such as KRAS, INSR, RPS6, CDK6 and PCK1. Several downstream effectors of the PI3K-AKT pathway regulate glycol-metabolism *via* GLUT and key rate-limiting enzymes in glycolysis, such as PFK1 and HK2, which are frequently elevated in CRC (17). In addition, it also activates downstream effectors that directly regulate cellular metabolic reprogramming, such as GSK3 (18). In fact, studies have indicated that loss of E-cadherin activates the PI3K/AKT signaling pathway resulting in the phosphorylation and inactivation of GSK3β, thus reducing degradation of β-catenin and promoting Wnt signaling (19). These findings indicate that EMT of CRC cells activates glycolysis. A recent study showed that INSR, a key link between insulin and PI3K/AKT signaling pathways, is also associated with the regulation of epithelial morphogenesis and differentiation (20). This suggests that the key proteins of glucose metabolism can also promote EMT. Furthermore, FA-mediated alteration of the DNA methylation levels of PI3K/AKT/CREB indirectly indicates that the expression level of FOLR affects the PI3K/AKT signaling pathway (21). Our results further suggest that FOLR regulates the PI3K/AKT signaling pathway by acting on INSR, and thus indirectly influences glucose metabolism as well. Furthermore, FA can activate FR-STAT3 signal transduction pathway (22), which promotes EMT through the PI3K/AKT signaling pathway (23). FOLR is overexpressed in some tumors and correlates significantly with the histological type, tumor grade, stage and lymph node metastasis (24). It is an established oncogenic marker and serum levels of FOLR can predict disease progression more stably and reliably compared to conventional tumor markers (25). Furthermore, since FOLR regulates the PI3K pathway through INSR, and acts as a bridge between tumor metabolic reprogramming and EMT, dual targeting of FOLR and INSR can monitor the occurrence, metastasis and prognosis of CRC with greater accuracy.

In addition, CEA can be used as a diagnostic indicator for colorectal cancer. CEA elevation is also an important reference indicator for disease progression and distant metastasis. In this paper, we have explored the mechanism of GMR and EMT for colorectal cancer, fully demonstrating the importance of glucose metabolism in colorectal cancer progression. Therefore, we innovatively combined CEA and glucose indicators to predict liver metastasis of colorectal cancer, and demonstrated certain advantages in diagnostic ability. CSR can be utilised as an innovative and highly specific indicator to distinguish whether liver metastasis in colorectal cancer patients. When CSR is used alone, not only the production of CEA in primary colorectal cancer, but also the glucose metabolism in distant liver metastases were considered. CSR can improve the sensitivity of liver metastasis in combination with other examination. Aberrant glucose metabolism drives EMT, whereas the latter can also reprogram the metabolic profile of cancer cells. Both biological phenomena are closely associated with malignant behaviors such as rapid proliferation, invasiveness and metastasis.

## Data availability statement

The datasets presented in this study can be found in online repositories. The names of the repository/repositories and accession number(s) can be found below: https://www.iprox.cn//page/SCV017.html?query=IPX0004385000.

## Ethics statement

The studies involving human participants were reviewed and approved by Guangxi Medical University Cancer Hospital. The patients/participants provided their written informed consent to participate in this study. Written informed consent was not obtained from the individual(s) for the publication of any potentially identifiable images or data included in this article.

## Author contributions

MH designed the study, led data analyses, and wrote the manuscript. LC and YW contributed to the analysis of the study and writing of the manuscript. LF and HY obtained clinical information, contributed to the design, experimental work and analysis. HR and XM obtained and documented clinical information. LY recruited patients, obtained blood samples and contributed to documentation of clinical information. ZS contributed to experimental design, critical discussion of the findings and to the final manuscript.

## Supplementary material

Supplementary Figure 1
**(A, B)** Clinical pathological sections of patients. **(C)** Correlation between serological data and glucose metabolism.

Supplementary Figure 2
**(A-F)** Driver genes or with mCRC datasets from previous studies. **(G)** CCND1 affects glucose metabolism in CRC similar to KRAS mutations. **(H)** CCND1 DNA copy number changes and infiltration of immune cells. **(I)** The T286I mutations was in particular an oncogenic hotspot.

Supplementary Figure 3
**(A)** Differentially expressed protein enriched in PI3K-Akt pathway. **(B)** peptide number and molecular weight of proteomics**. (C, D)** Differences in protein expression levels among the different groups.

Supplementary Figure 4
**(A)** Principal component analyses (PCA) of proteomics. **(B-D)** Differentially expressed protein about the KEGG and GO enrichment analysis.

Supplementary Figure 5 Antifolate resistance, Endocytosis and PI3K-AKT pathway of KEGG enrichment analysis.

Supplementary Figure 6
**(A)** The FOLR2-CDK6-INSR axis was further analyzed by LC-MS/MS in the PRM mode. **(B)** The retention time of inner-label iRT was stable and the error was small. **(C)** Comparison in groups of KRAS, PCK1, INSR, CDK6 and RPS6.

Supplementary Figure 7
**(A)** 5 key mutant gene (KRAS, CCND1, PTEN, MTOR and TP53) and 14 functional states (occurrence, metastasis, treatment etc.) of CRC. **(B)** Functional correlation of TP53 and CCND1 was established in 290 cells. **(C)** T-SNE analysis further revealed uniform distribution of CCND1, PTEN, MTOR, TP53, INSR, CDK6, RPS6 and PCK1. **(D)** Immune infiltration about KRAS, TP53 and PTEN. **(E)** Tumor immune cells expressing GMR/EMT-related factors are prognostically relevant in CRC.

Supplementary Figure 8 GMR in CRC were identified by comparing the proteomic alterations in paired Ca, P and Liver samples from 12 mCRC patients.

Supplementary Figure 9 Disease free survival (DFS) and overall survival (OS) of CDK6, EPCAM and CCND1.

Supplementary Table 1 Baseline of clinical data of patients.

Supplementary Table 2 Patient serological data.

Supplementary Table 3 Differentially expressed genes (DEGs) related to metabolic functions.

Supplementary Table 4 Mutated genes enriched in the PI3K-AKT signaling pathway.
