# Supplementary material for: Multi-omics analyses of glucose metabolic reprogramming in colorectal cancer
Source: Front Immunol. 2023 Jul 5;14:1179699. doi: 10.3389/fimmu.2023.1179699 (PMC10354426; doi:10.3389/fimmu.2023.1179699)

**Supplementary Figure 1**


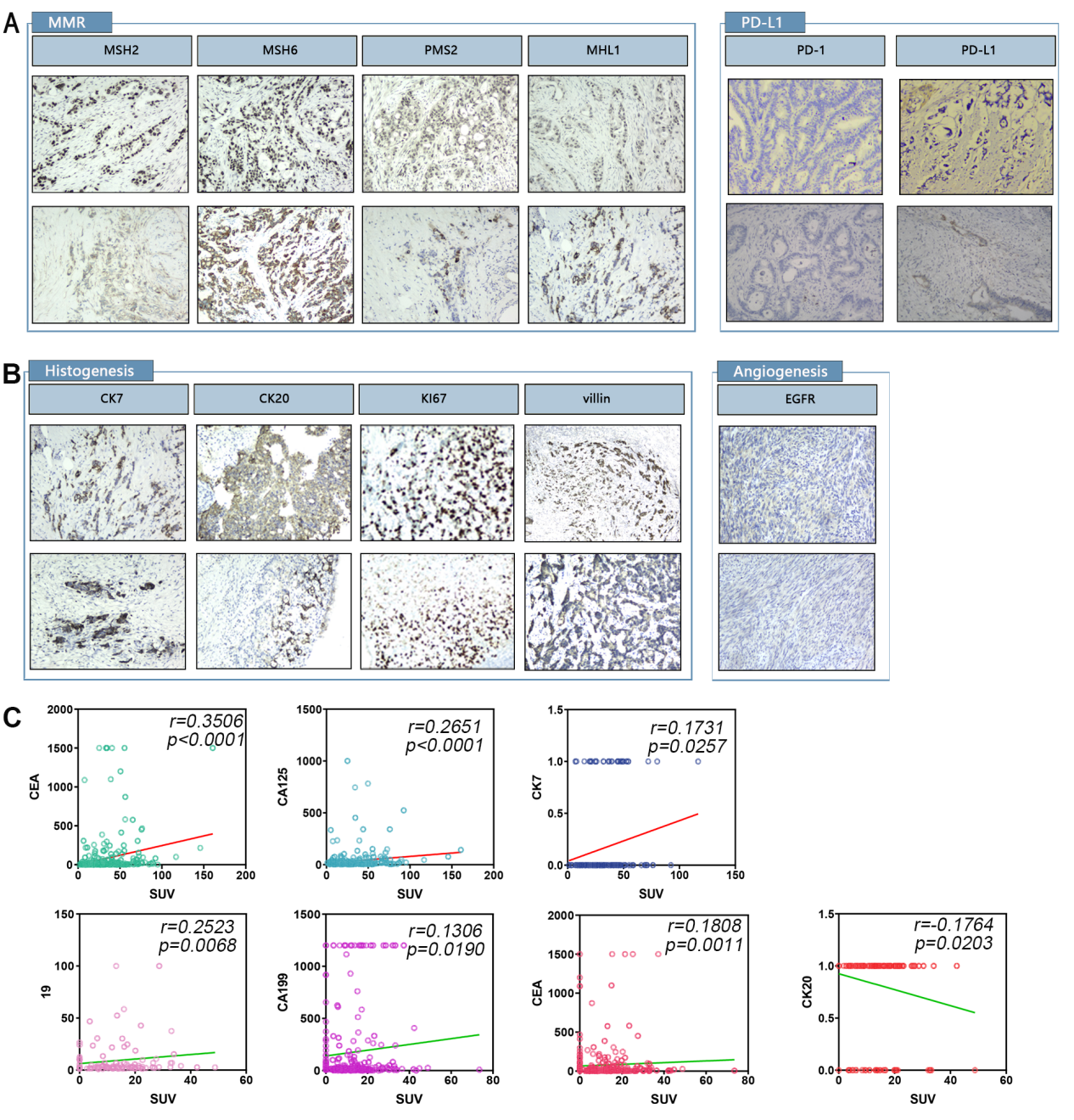


**Supplementary Figure 2**


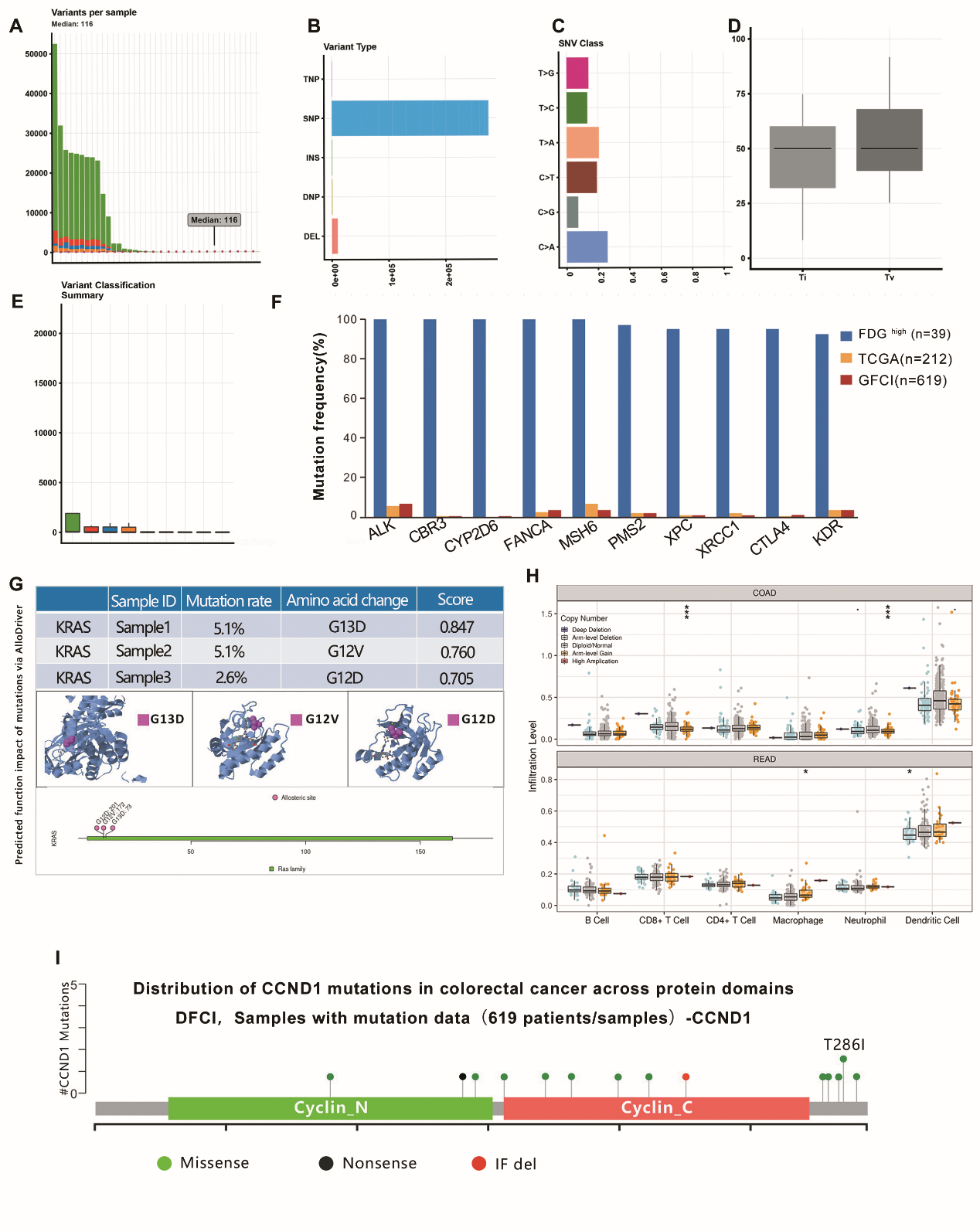


**Supplementary Figure 3**


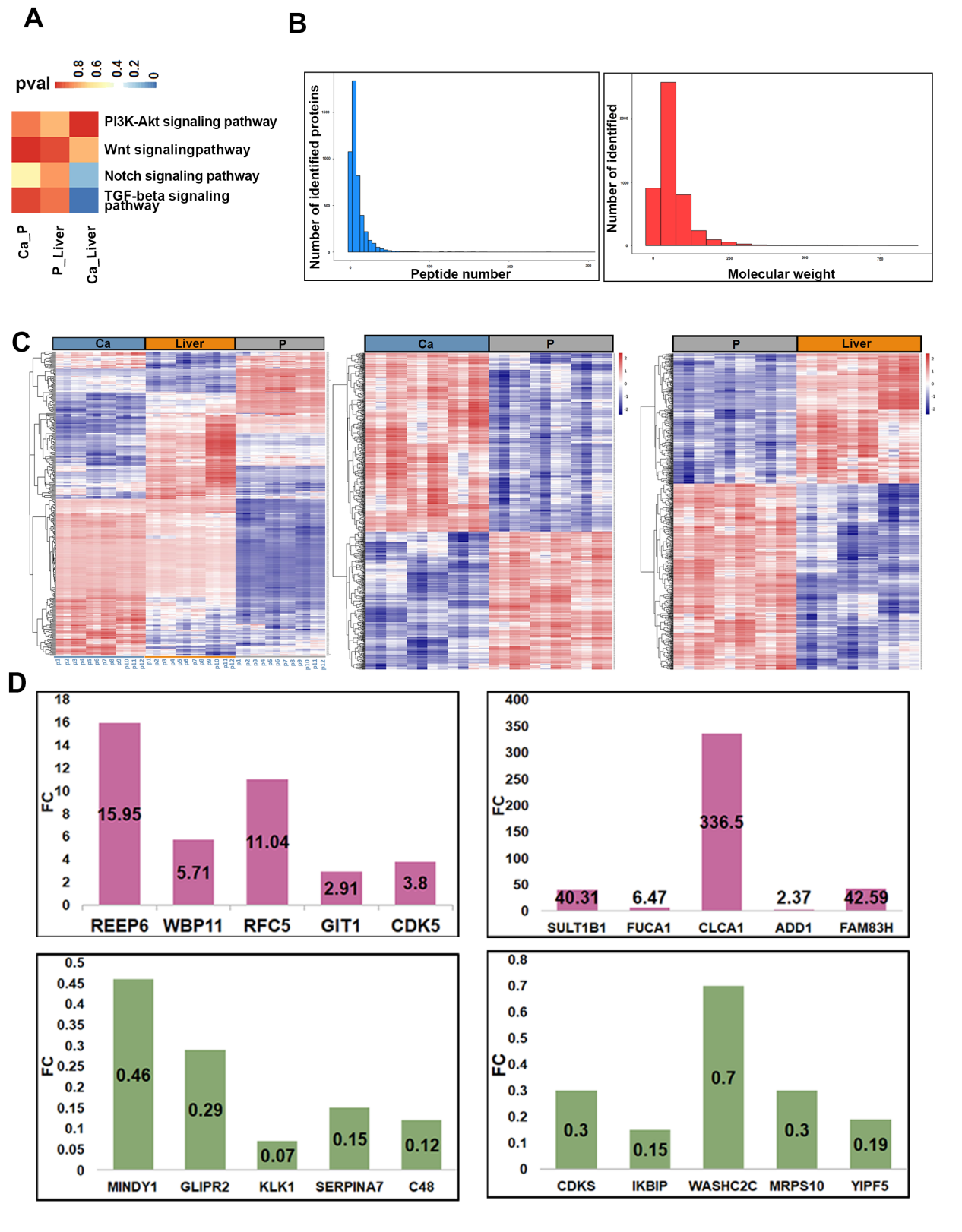


**Supplementary Figure 4**


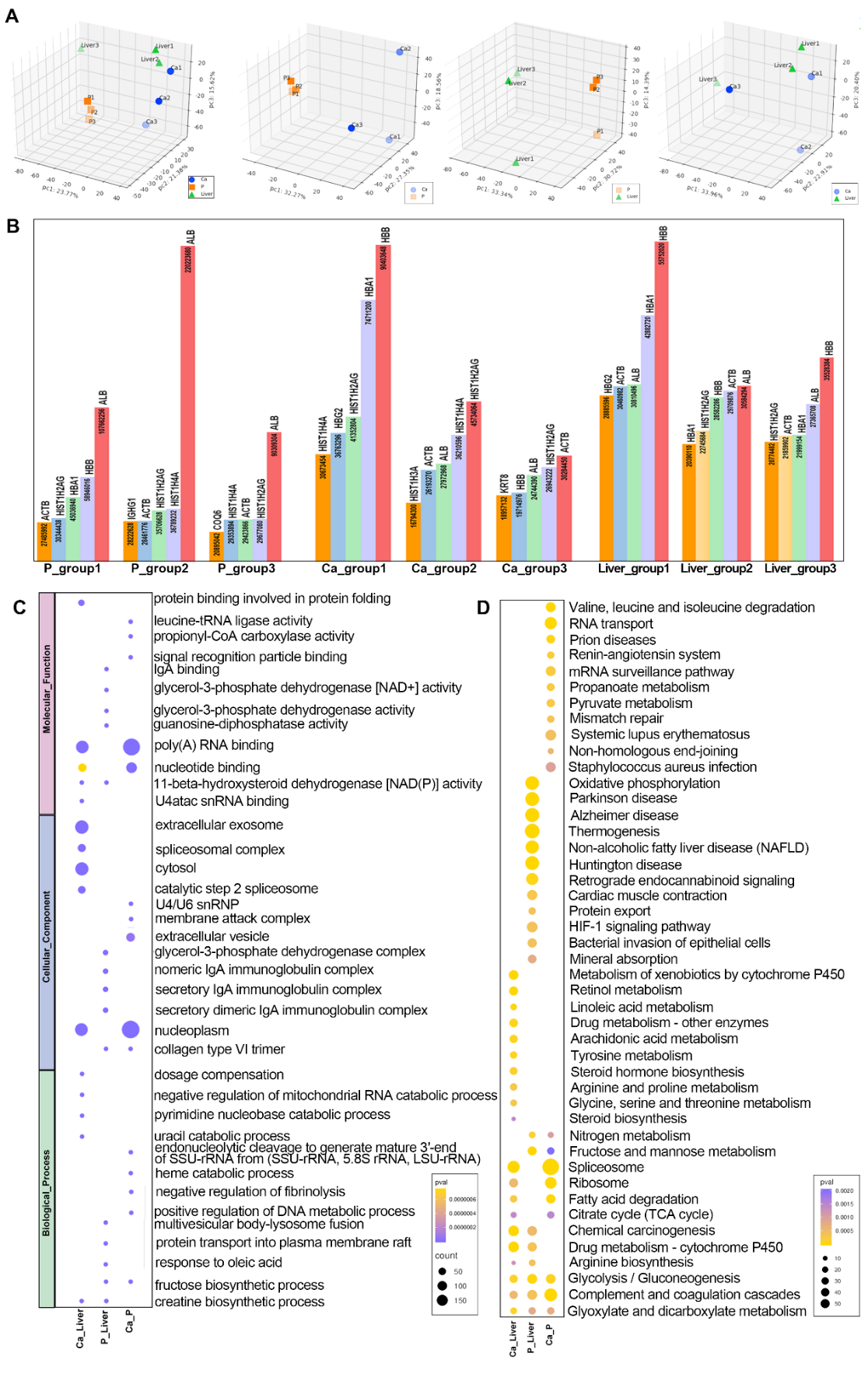


**Supplementary Figure 5**


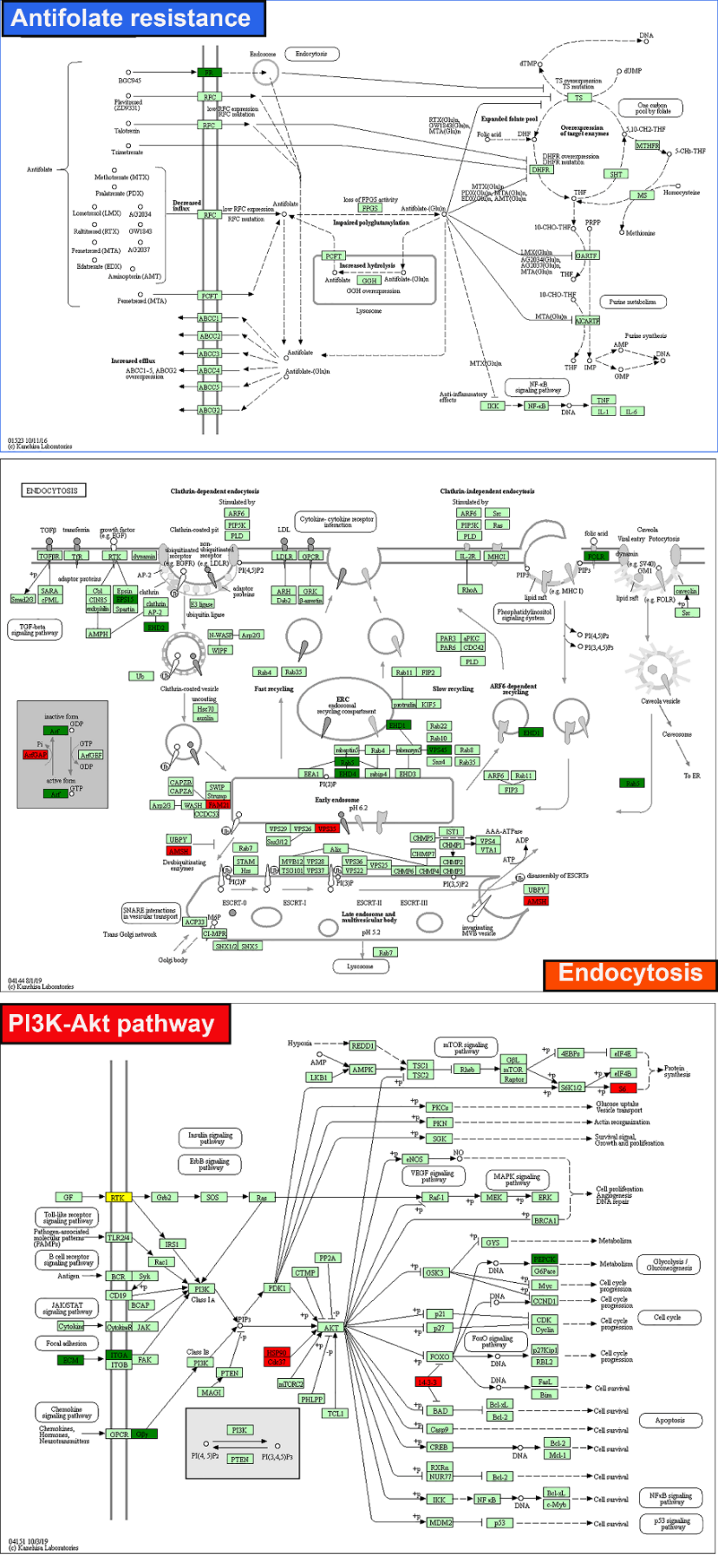


**Supplementary Figure 6**


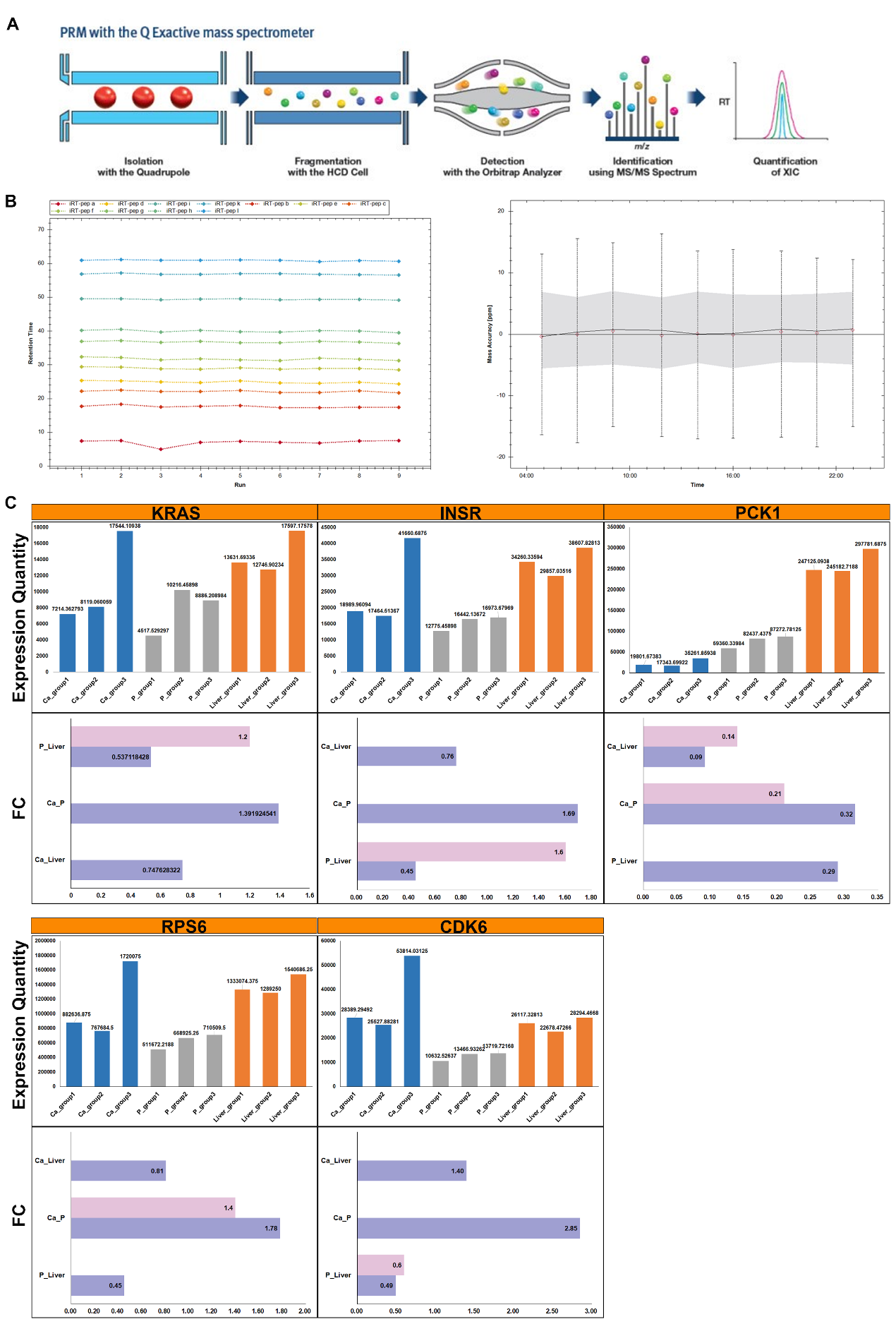


**Supplementary Figure 7**


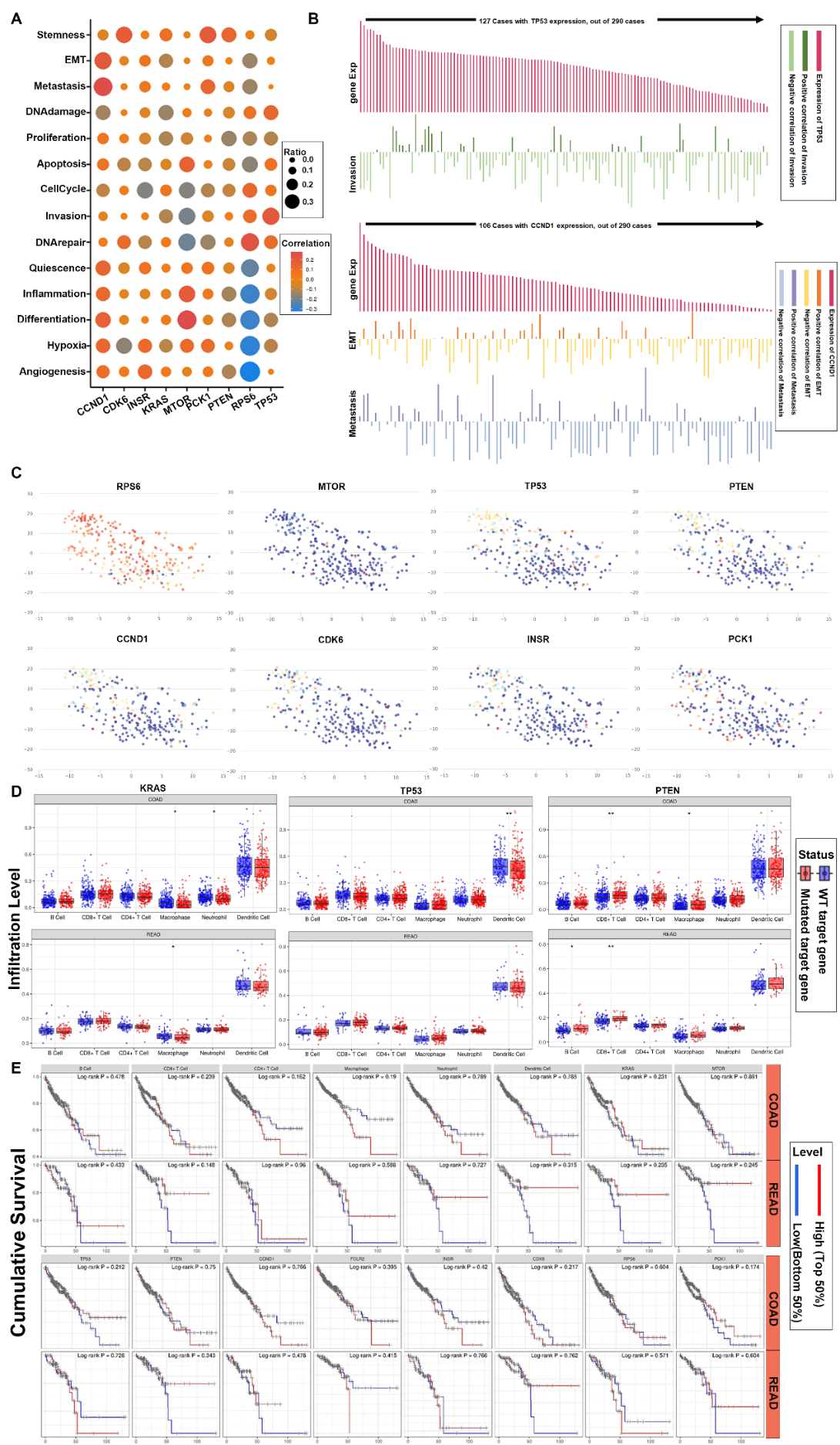


**Supplementary Figure 8**


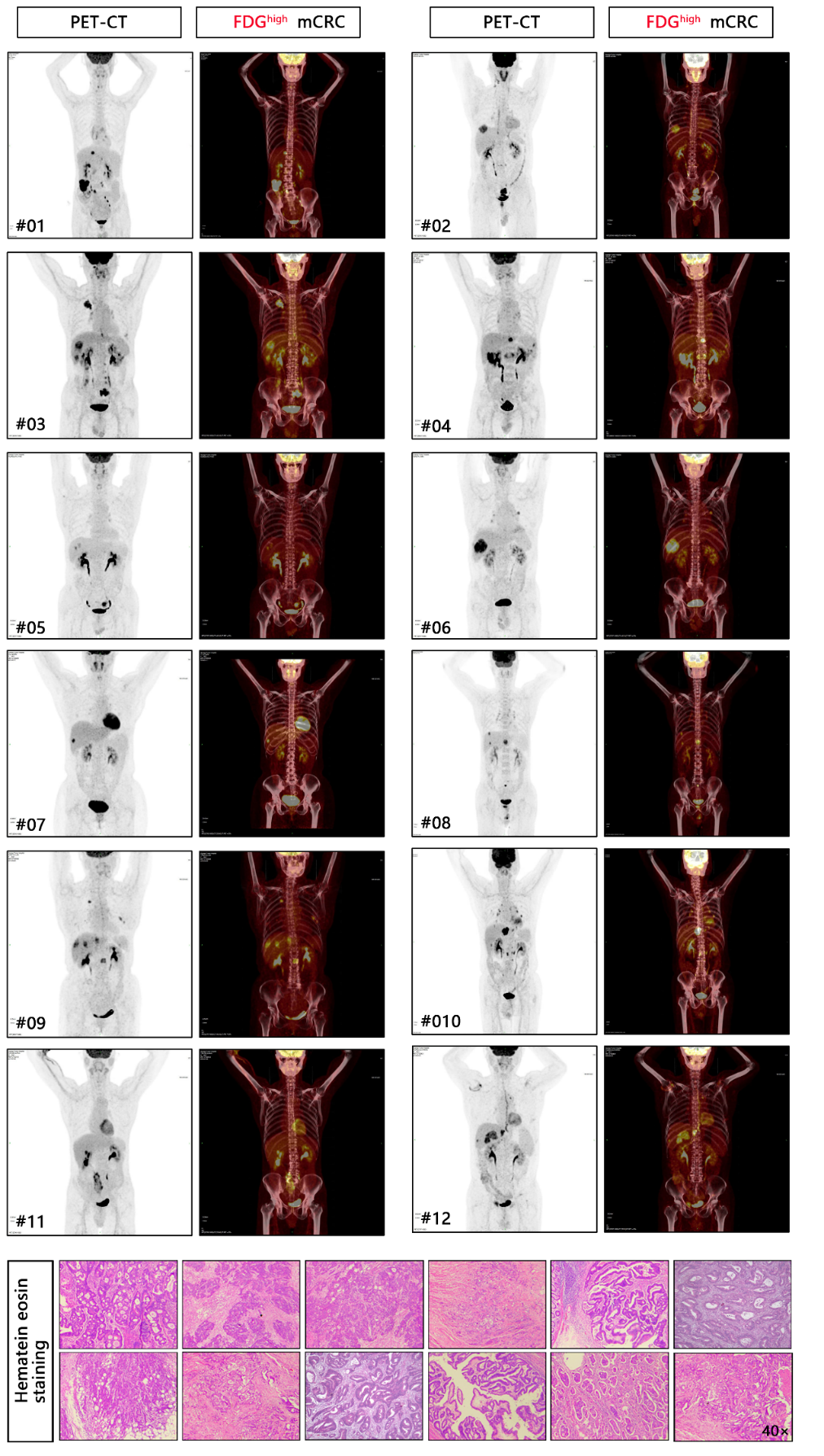


**Supplementary Figure 9**


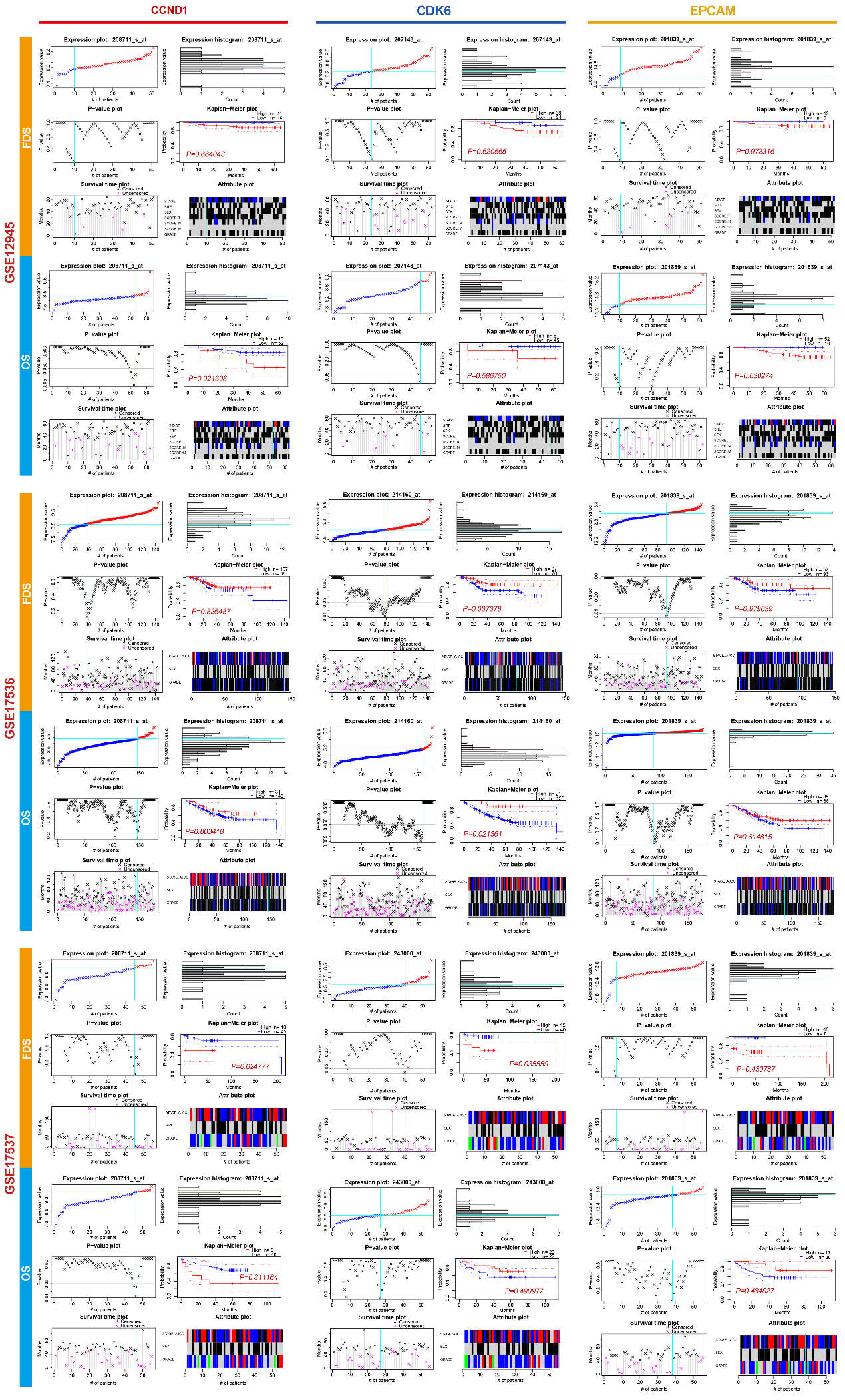

Supplement: Supplementary Figure 1 — (A, B) Clinical pathological sections of patients. (C) Correlation between serological data and glucose metabolism. [file DataSheet_1.docx]
