# Supplementary material for: Multi-omics analyses of glucose metabolic reprogramming in colorectal cancer
Source: Front Immunol. 2023 Jul 5;14:1179699. doi: 10.3389/fimmu.2023.1179699 (PMC10354426; doi:10.3389/fimmu.2023.1179699)
Supplement: Supplementary Table 1 — Baseline of clinical data of patients. [file Table_1.docx]

**Supplementary table 1**

| **Characteristic** | **Number of cases (Percentage of total)** |
| --- | --- |
| **CK7** |  |
| Positive | 26(15.66) |
| Negative | 140(84.34) |
| **CK20** |  |
| Positive | 147(84.97) |
| Negative | 26(15.03) |
| **Villin** |  |
| Positive | 130(97.01) |
| Negative | 4(2.99) |
| **β-catenin** |  |
| Positive | 52(42.98) |
| Negative | 69(57.02) |
| **S100** |  |
| Positive | 60(61.22) |
| Negative | 38(38.78) |
| **P53** |  |
| Positive | 110(83.33) |
| Negative | 22(16.67) |
| **Ki67** |  |
| Positive | 184(99.46) |
| Negative | 1(0.54) |
| **MLH1** |  |
| Positive | 193(98.47) |
| Negative | 3(1.53) |
| **PMS2** |  |
| Positive | 191(97.45) |
| Negative | 5(2.55) |
| **MSH1** |  |
| Positive | 193(98.47) |
| Negative | 3(1.53) |
| **MSH6** |  |
| Positive | 191(97.45) |
| Negative | 5(2.55) |

**Supplementary table 2**

| **Characteristic** | **Number of cases (Percentage of total)** |
| --- | --- |
| **Gender** |  |
| Male | 180 (55.21) |
| Female | 146(44.79) |
| **Age/year** |  |
| ＜60 | 172(55.76) |
| ≥60 | 154(47.24) |
| **CEA** |  |
| Abnormal | 172(52.76) |
| Normal | 154(47.24) |
| **AFP** |  |
| Abnormal | 4(1.23) |
| Normal | 322(98.77) |
| **CA125** |  |
| Abnormal | 55(16.87) |
| Normal | 271(83.13) |
| **CA153** |  |
| Abnormal | 13(3.99) |
| Normal | 313(96.01) |
| **CA199** |  |
| Abnormal | 99(30.37) |
| Normal | 227(69.63) |
| **Blood sugar** |  |
| Abnormal | 62(19.02) |
| Normal | 264(80.98) |
| **Total bilirubin** |  |
| Abnormal | 46(14.11) |
| Normal | 280(85.89) |
| **ALT** |  |
| Abnormal | 23(7.06) |
| Normal | 303(92.94) |
| **AST** |  |
| Abnormal | 29(8.90) |
| Normal | 297(91.10) |
| **Creatinine** |  |
| Abnormal | 12(3.68) |
| Normal | 314(96.32) |
| **Urea** |  |
| Abnormal | 80(24.54) |
| Normal | 246(75.46) |
| **Cholesterol** |  |
| Abnormal | 113(34.66) |
| Normal | 213(35.34) |
| **Triglycerides** |  |
| Abnormal | 65(19.94) |
| Normal | 261(80.06) |
| **HDL** |  |
| Abnormal | 17(5.21) |
| Normal | 309(94.79) |
| **LDL** |  |
| Abnormal | 183(56.13) |
| Normal | 143(46.87) |

.

supplement table 3

| **Signaling pathways** | **genes** |
| --- | --- |
| Metabolic | \| POLD1 \| IDH2 \| SDHB \| TP53 \| CTNNB1 \| \| --- \| --- \| --- \| --- \| --- \| \| POLE \| SDHC \| SDHD \| STK11 \| PTEN \| \| MTOR \| GNAQ \| FBXW7 \| PIK3CA \| ARID1A \| \| PIK3R1 \| GNAS \| IDH1 \| AKT1 \| TSC2 \| \| MYC \| NFE2L2 \| SMARCA4 \| CCND1 \| TSC1 \| |
| Proliferation | \| CCND1 \| CTNNB1 \| ATRX \| ERBB3 \| AKT1 \| \| --- \| --- \| --- \| --- \| --- \| \| TP53 \| ESR1 \| U2AF1 \| DDR2 \| TSC2 \| \| PIK3CA \| VHL \| RAD50 \| ERBB2 \| MAP2K2 \| \| FGFR4 \| RUNX1 \| CHEK1 \| RET \| FGFR3 \| \| MYC \| AR \| BRCA2 \| ROS1 \| STK11 \| \| JAK2 \| TERT \| RAD51C \| NTRK2 \| NRAS \| \| CDK4 \| APC \| MSH6 \| PTPN11 \| EGFR \| \| BRAF \| GNAS \| KDM5C \| ALK \| CDK6 \| \| FGFR1 \| PTCH1 \| CHEK2 \| MAX \| TMEM127 \| \| KRAS \| ARAF \| MYCN \| GNAQ \| FGFR2 \| \| TSC1 \| ERBB4 \| FBXW7 \| RB1 \| JAK3 \| \| KIT \| NF1 \| NOTCH1 \| NFE2L2 \| KDR \| \| PIK3R1 \| NTRK3 \| MEN1 \| KDM6A \| MET \| \| PTEN \| FLT3 \| POLE \| POLD1 \| BRCA1 \| \| MDM2 \| LRP1B \| MRE11A \| CDK12 \| MTOR \| \| MYD88 \| NTRK1 \| CDH1 \| BMPR1A \| SMAD4 \| \| SMARCA4 \| MSH2 \| CDKN2A \| PMS2 \| MLH1 \| \| PALB2 \| FANCC \| BARD1 \| MUTYH \| FANCA \| \| NPM1 \| SF3B1 \| PDGFRA \| HRAS \| MAP2K1 \| \| RAF1 \| ATM \|  \|  \|  \| |
| Microenvironment | \| KRAS \| TSC1 \| PIK3CA \| CCND1 \| AKT1 \| \| --- \| --- \| --- \| --- \| --- \| \| RAF1 \| MTOR \| PIK3R1 \| MYC \| MYCN \| \| RIT1 \| KDM5C \| GNAQ \| HOXB13 \| PTPN11 \| \| NRAS \| KDM6A \| PTCH1 \| JAK2 \| CDH1 \| \| MAP2K1 \| MEN1 \| GNAS \| JAK3 \| VHL \| \| HRAS \| EGFR \| SMO \| SMARCA4 \| APC \| \| MAP2K2 \| PBRM1 \| PTEN \| ARID1A \| EPCAM \| \| CDK4 \| AR \| TSC2 \| BAP1 \| KDR \| \| MYD88 \| ESR1 \|  \|  \|  \| |

supplement table 4

| node1 | node2 | node1_string_internal_id | node2_string_internal_id | node1_external_id | node2_external_id | neighborhood_on_chromosome | gene_fusion | phylogenetic_cooccurrence | homology | coexpression | experimentally_determined_interaction | database_annotated | automated_textmining | combined_score |
| --- | --- | --- | --- | --- | --- | --- | --- | --- | --- | --- | --- | --- | --- | --- |
| PTEN | TP53 | 4443848 | 4435880 | 9606.ENSP00000361021 | 9606.ENSP00000269305 | 0 | 0 | 0 | 0 | 0 | 0.472 | 0.9 | 0.963 | 0.997 |
| PTEN | MTOR | 4443848 | 4442490 | 9606.ENSP00000361021 | 9606.ENSP00000354558 | 0 | 0 | 0 | 0 | 0 | 0.314 | 0 | 0.932 | 0.951 |
| PTEN | KRAS | 4443848 | 4434456 | 9606.ENSP00000361021 | 9606.ENSP00000256078 | 0 | 0 | 0 | 0 | 0.063 | 0.186 | 0 | 0.882 | 0.902 |
| PTEN | CCND1 | 4443848 | 4433391 | 9606.ENSP00000361021 | 9606.ENSP00000227507 | 0 | 0 | 0 | 0 | 0 | 0 | 0 | 0.893 | 0.893 |
| MTOR | TP53 | 4442490 | 4435880 | 9606.ENSP00000354558 | 9606.ENSP00000269305 | 0 | 0 | 0 | 0 | 0 | 0.391 | 0 | 0.835 | 0.895 |
| MTOR | CCND1 | 4442490 | 4433391 | 9606.ENSP00000354558 | 9606.ENSP00000227507 | 0 | 0 | 0 | 0 | 0.062 | 0 | 0 | 0.804 | 0.809 |
| MTOR | KRAS | 4442490 | 4434456 | 9606.ENSP00000354558 | 9606.ENSP00000256078 | 0 | 0 | 0 | 0 | 0.061 | 0.322 | 0 | 0.725 | 0.809 |
| TP53 | CCND1 | 4435880 | 4433391 | 9606.ENSP00000269305 | 9606.ENSP00000227507 | 0 | 0 | 0 | 0 | 0.061 | 0.379 | 0 | 0.942 | 0.963 |
| TP53 | KRAS | 4435880 | 4434456 | 9606.ENSP00000269305 | 9606.ENSP00000256078 | 0 | 0 | 0 | 0 | 0 | 0 | 0 | 0.924 | 0.924 |
| KRAS | CCND1 | 4434456 | 4433391 | 9606.ENSP00000256078 | 9606.ENSP00000227507 | 0 | 0 | 0 | 0 | 0 | 0.078 | 0 | 0.828 | 0.836 |

**Supplement table 5**

**Comparisons of parameter between the patients of colorectal cancer of liver metastasis and non-liver metastases in discovery cohort**

| Parameter | liver metastases  N=99 (means ± SEM) | Non- liver metastases  N=65 (means ± SEM) |
| --- | --- | --- |
| Age, years | 58.25±1.13 | 58.78±1.59 |
| Gender (M / F) | 56 / 43 | 36 / 29 |
| RBC | 4.25±0.07 | 4.28±0.09 |
| WBC | 6.86±0.24 | 6.78±0.27 |
| Hb | 116.6±2.36 | 115.4±3.07 |
| SUV of liver | 7.81±0.76 * * * * | 0.23±0.17 * * * * |
| CEA | 128.9±30.15 * * * * | 14.51±4.44 * * * * |
| CA199 | 311±46.64 * * * * | 49.05±20.11 * * * * |
| CA125 | 41.44±11.28 * * | 14.49±2.31 * * |
| CA153 | 12.03±1.34 | 9.48±0.59 |
| AFP | 2.61±0.12 | 3.49±0.60 |
| Blood sugar | 4.82±0.11 | 4.79±0.17 |
| CEA to blood sugar ratio (CSR) | 27.43±6.899 * * * * | 2.96±0.86 * * * * |

* *p*＜0.05 between hepatic metastases and non-hepatic metastases

* * *p*＜0.01 between hepatic metastases and non-hepatic metastases

* * *  *p*＜0.001 between hepatic metastases and non-hepatic metastases

* * * * *p*＜0.0001 between hepatic metastases and non-hepatic metastases

**Supplement table 6**

**Diagnostic value of alone and combined biomarkers for distinguishing mCRC patients in discovery cohort**

| Variables | AUC | *p* value | Cut-off | Sensitivity | Specificity | 95% Confidence interval | |
| --- | --- | --- | --- | --- | --- | --- | --- |
|  |  |  |  |  |  | Upper limit | Lower limit |
| CEA | 0.748 | <0.0001 | 19.610 | 52.530 | 87.690 | 0.673 | 0.822 |
| CA199 | 0.676 | <0.001 | 93.300 | 40.400 | 93.850 | 0.595 | 0.757 |
| CSR | 0.753 | <0.0001 | 5.025 | 47.920 | 90.630 | 0.678 | 0.828 |
| Blood sugar | 0.526 | 0.586 | 29.650 | 18.750 | 98.440 | 0.435 | 0.616 |
| SUV of liver | 0.815 | <0.0001 | 2.750 | 65.660 | 98.460 | 0.750 | 0.880 |
| SUV of liver + CEA | 0.871 | <0.0001 | - | 67.680 | 98.460 | 0.818 | 0.925 |
| SUV of liver + Blood sugar | 0.830 | <0.0001 | - | 64.580 | 98.440 | 0.767 | 0.893 |
| SUV of liver + CSR | 0.878 | <0.0001 | - | 69.790 | 96.880 | 0.813 | 0.922 |
| CA199+CSR | 0.781 | <0.0001 | - | 63.540 | 81.250 | 0.711 | 0.851 |

**Supplement table 7**

**Diagnostic value of alone biomarkers for distinguishing mCRC patients without diabetes in discovery cohort**

| Variables | AUC | *p* value | Cut-off | Sensitivity | Specificity | 95% Confidence interval | |
| --- | --- | --- | --- | --- | --- | --- | --- |
|  |  |  |  |  |  | Upper limit | Lower limit |
| CEA | 0.7499 | <0.0001 | 19.610 | 53.490 | 86.890 | 0.6720 | 0.8278 |
| Blood sugar | 0.5016 | 0.9733 | 47.700 | 34.880 | 95.080 | 0.4067 | 0.5966 |
| CSR | 0.7528 | <0.0001 | 5.025 | 48.840 | 96.160 | 0.6753 | 0.8302 |

**Supplement table 8**

**Comparisons of parameter between the patients of colorectal cancer of liver metastasis and non-hepatic metastases in validation cohort**

| Parameter | liver metastases  N=99 (means ± SEM) | Non- liver metastases  N=65 (means ± SEM) |
| --- | --- | --- |
| Age, years | 54.35±1.53 | 59.48±1.80 |
| Gender (M / F) | 34 / 31 | 27 / 13 |
| CEA | 202.4±53.40 * * * * | 13.90±7.31 * * * * |
| Blood sugar | 5.32±0.27 | 5.53±0.44 |
| CEA to blood sugar ratio (CSR) | 38.35±10.82 * * * * | 3.09±1.67 * * * * |

* *p*＜0.05 between hepatic metastases and non-hepatic metastases

* * *p*＜0.01 between hepatic metastases and non-hepatic metastases

* * *  *p*＜0.001 between hepatic metastases and non-hepatic metastases

* * * * *p*＜0.0001 between hepatic metastases and non-hepatic metastases

**Supplement table 9**

**Diagnostic value of alone biomarkers for distinguishing mCRC patients in validation cohort**

| Variables | AUC | *p* value | Cut-off | Sensitivity | Specificity | 95% Confidence interval | |
| --- | --- | --- | --- | --- | --- | --- | --- |
|  |  |  |  |  |  | Upper limit | Lower limit |
| CEA | 0.7990 | <0.0001 | 6.345 | 67.690 | 82.500 | 0.713 | 0.884 |
| Blood sugar | 0.5440 | 0.452 | 4.880 | 46.150 | 72.500 | 0.423 | 0.660 |
| CSR | 0.8040 | <0.0001 | 3.090 | 53.850 | 95.000 | 0.719 | 0.889 |
